# *Mycobacterium goodii* Infections Associated with Surgical Implants at Colorado Hospital

**DOI:** 10.3201/eid1010.040402

**Published:** 2004-10

**Authors:** Dayna Devon Ferguson, Ken Gershman, Bette Jensen, Matthew J. Arduino, Mitchell A. Yakrus, Robert C. Cooksey, Arjun Srinivasan

**Affiliations:** * Colorado Department of Public Health and Environment, Denver, Colorado, USA;; † Centers for Disease Control and Prevention, Atlanta, Georgia, USA

**Keywords:** Mycobacterium goodii, atypical mycobacteria, nosocomial infection, postoperative wound infection, implants, dispatch

## Abstract

From February to October 2003, *Mycobacterium goodii* wound infections were identified among three patients who received surgical implants at a Colorado hospital. This report summarizes the investigation of the first reported nosocomial outbreak of *M. goodii*. Increased awareness is needed about the potential for nontuberculous mycobacteria to cause postoperative wound infections.

*Mycobacterium goodii* is a recently identified, rapidly growing nontuberculous mycobacteria species of the *M. smegmatis* group (*1*). Previously associated with sporadic cases of cellulitis, osteomyelitis, infected pacemaker sites, lipoid pneumonia (*1**,**2*), and bursitis (*3*), *M. goodii* has not been associated with outbreaks.

## The Study

In June 2003, staff at hospital A contacted the Centers for Disease Control and Prevention (CDC) and the Colorado Department of Public Health and Environment (CDPHE) about two patients (patients 2 and 3) in whom *M. goodii* wound infections developed after surgery at hospital A. In October 2003, patient 1 notified CDPHE that he had a *M. goodii* wound infection; he had previously undergone surgery at hospital A.

### Patient 1

A 64-year-old man underwent left total hip arthroplasty at hospital A in April 2002. In May 2003, he was evaluated at hospital A for chronic left hip pain. Bone scan suggested prosthesis loosening or infection. Gram stain and bacterial culture of aspirated joint fluid were negative. Mycobacterial culture was not performed.

In July 2003, he underwent arthroplasty revision at hospital B for persistent pain. A preoperative sedimentation rate was normal, but a substantial amount of yellow fluid was noted during operative manipulation of the trochanteric bursa. Fluid Gram stain demonstrated a moderate number of leukocytes without organisms; bacterial culture was negative, and mycobacterial culture was not performed.

Wound erythema with drainage developed 4 weeks later, and septic hip arthritis was diagnosed at hospital B, requiring prosthesis removal. Joint fluid, decontaminated with Sputagest 50 mucolytic agent (Remel, Lenexa, KS), grew mycobacteria after 7 days of incubation at 36°C with Middlebrook 7H12 TB media (Becton Dickinson, Sparks, MD) and Lowenstein-Jensen agar. Resulting colonies were smooth, flat, mucoid, brownish-orange and later identified as *M. goodii* sensitive to ciprofloxacin (MIC = 1 µg/mL), doxycycline (MIC < 0.5 µg/mL), and trimethoprim/sulfamethoxazole (MIC = 1 µg/mL).

### Patient 2

A 64-year-old man underwent right inguinal hernia repair with a Kugel patch at hospital A in January 2003. On postoperative day 17, he underwent elective L3–L4 laminectomy at hospital C. Right inguinal pain, swelling, and erythema developed 2 days later, and he required wound debridement and patch removal. Patch cultures grew mycobacteria after 3 days of incubation at 35°C with sheep blood agar and thioglycolate broth without pretreatment. The isolate was later identified as *M. goodii* sensitive to ciprofloxacin (MIC = 0.25 µg/mL), doxycycline (MIC < 0.5 µg/mL), and trimethoprim/sulfamethoxazole (MIC = 2 µg/mL).

### Patient 3

A woman 75 years of age underwent a total replacement of her left knee at hospital A in April 2003. On postoperative day 14, pain and swelling developed in her left knee. Fluid aspirated from the wound on postoperative day 29 was negative by Gram stain and bacterial culture. Mycobacterial culture was not performed, and antimicrobial drugs were withheld. Repeat Gram stain 9 days later demonstrated many leukocytes without organisms. The prosthesis was later removed and replaced with a stabilizing polyethylene insert. Wound drainage samples, decontaminated with N-acetyl-L-cysteine-sodium hydroxide, grew mycobacteria after 11 days of incubation at 37°C with BacT/ALERT media (bioMérieux, Durham, NC) and Lowenstein-Jensen agar. The isolate was later identified as *M. goodii* sensitive to ciprofloxacin (MIC = 0.25 µg/mL), doxycycline (MIC < 0.5 µg/mL), and trimethoprim/sulfamethoxazole (MIC = 4 µg/mL).

Isolates were confirmed as *M. goodii* with 65-kD heat shock protein gene polymerase chain reaction restriction analysis, by using enzymes *Bst*EII, *Hae*III, *Bsa*HI, and *Aci*I (New England Biolabs, Beverly, MA) and matching them to a control strain, *M. goodii* ATCC 700504 (*1*). Susceptibilities were assessed according to the NCCLS document M24-A (*4*). Isolates were grown and compared by pulsed-field gel electrophoresis (PFGE) according to previously described methods (*5*). Genomic DNA restriction was performed with 40 U of *Ase*I (New England Biolabs, Beverly, MA). Less than a three-band difference was found among the resulting patterns (Figure). Based on previously described criteria (*6*), these isolates were closely related and probably from the same source.

**Figure:**
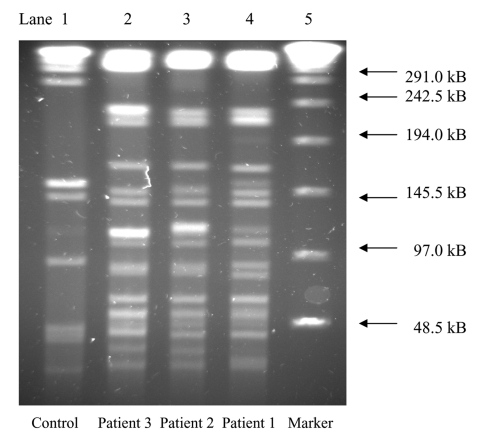
Pulsed-field gel electrophoresis patterns using restriction enzyme *Ase*I, of *Mycobacterium goodii* isolates from three patients with postoperative wound infections after receiving surgical implants from a hospital in Colorado. Lane 1 is control strain *M. goodii* ATCC 700504. Lanes 2–4 are case-isolates. Lane 5 is a 48.5-kb DNA marker.

Anesthesiologist A was the only person who had contact with all three patients during an operative procedure (Table). Anesthesiologist A was also present during an operation on a potential case-patient in whom an inguinal wound infection developed 29 days after his hernia had been repaired with a Kugel patch at hospital A in January 2003. Although mycobacterial cultures were not performed, a Gram stain of drainage material showed many leukocytes without organisms, and bacterial cultures were negative. This patient improved after surgical drainage, patch removal, and treatment with cephalexin. No other potential cases were identified retrospectively through questioning of surgical staff or prospectively with active surveillance.

**Table:** Summary of investigation of three cases of *Mycobacterium goodii* postoperative wound infections associated with surgical implants at a Colorado hospital

Criteria	Patient 1	Patient 2	Patient 3
Age	64	64	75
Sex	Male	Male	Female
Date of surgery	4/02	1/03	4/03
Date infection diagnosed	8/03 (16 mo after surgery)	2/03 (19 d after surgery)	5/03 (29 d after surgery)
Initial surgery	Total hip arthroplasty	Inguinal hernia repair	Total knee replacement
Hospital^a^	A	A	A
Surgical implant	Artificial hip	Kugel patch	Artificial knee
Operating room (OR)^b^	A	B	A
Surgeon^c^	A	B	C
Anesthesiologist^d^	A	A	A
Assistant^e^	A	None	A, B
OR personnel^f^	A, B, C, D	A, E	B, F, G, H
Skin preparation	Povidone-iodine, isopropyl alcohol	Povidone-iodine, ethanol	Povidone-iodine, isopropyl alcohol
Local anesthetic	Bupivicaine	Bupivicaine	None
Saline irrigation	Yes (with kanamycin)	Yes	Yes
Intraoperative antibiotics	Cefazolin	None	Cefazolin
Notes	Hip aspiration, hospital A, 5/03; Hip arthroplasty revision, hospital B, 7/03	Laminectomy, hospital C, 2/03	

^a^The three patients received implants at hospital A. ^b^The three patients had surgery in two operating rooms (A and B) at hospital A. ^c^Three surgeons (A-C) were involved in these three operations at hospital A. ^d^One anesthesiologist (A) was involved in the three operations at hospital A. ^e^Two different assistants (A and B) were involved in two of the three operations at hospital A. ^f^Includes circulating nurses, scrub technicians, a surgical sales representative and any staff not previously listed who were working in the operating rooms during these operations (A-H).

Anesthesiologist A was assessed for *M. goodii* carriage by collecting a sputum sample, beard clippings, and sampling his nares and operating shoes with sterile swabs. Swabs were placed in Butterfield buffer (Becton Dickinson, Cockeyville, MD), exposed to 0.005% cetylpyridinium chloride monohydrate, vortexed, concentrated, added to 7H10 agar plates, and incubated at 35°C and 30°C for 14 days. Hand screening was also performed by using sterile premoistened wipes to wipe both hands and each finger. Wipes were processed and cultured similar to the procedure described above for swabs. All specimens from anesthesiologist A were negative.

Specimens from hands and operating room shoes were also collected from 12 staff members present in operating rooms before or during any of the three patients' procedures. All of these specimens were negative.

Water was tested by using sterile swabs to sample biofilm and water from faucets in the operating room area, including the scrub, sterile supply, and clean and dirty utility room sinks. Sterile swabs were also used to sample lights, vents, and anesthesia tables in the operating rooms, equipment sterilizers, and rubber floor mats adjacent to scrub sinks. All environmental samples were negative. No environmental breaches were found in the sterile supply area, including no evidence of water damage, and no recent construction.

Two patients received local anesthesia from single dose vials during surgery. Alcohol and povidone-iodine were used for skin preparation before all three procedures, and quaternary ammonium was used to disinfect operating rooms at hospital A. Clinical laboratories in Colorado were contacted, and no other *M. goodii* cases were identified in Colorado from 2002 to 2003.

## Conclusions

Although this cluster occurred over an extensive period, it likely had a common etiology because *M. goodii* has been infrequently identified as a pathogen, and isolates had closely-related PFGE patterns. PFGE has been reported as a means of identifying outbreaks of other nontuberculous mycobacteria, including *M. fortuitum* (*5*), *M. chelonae*, and *M. abscessus* (*7*).

Additionally, all patients received surgical implants at the same hospital before their infections. Although patient 1 had arthroplasty revision at another hospital 1 month before diagnosis, chronic hip pain, an abnormal bone scan before revision, and an abnormal amount of bursal fluid during surgery suggest that he had indolent infection that preceded the second hip surgery.

Anesthesiologist A seemed a possible infection source because he was the only person present during each surgery at hospital A. Unscrubbed surgical personnel have been linked to other postoperative wound infections, including an anesthesiologist colonized with *Nocardia farcinica* who reportedly caused five postoperative sternotomy infections (*8*). Although our investigation did not identify anesthesiologist A as an *M. goodii* carrier, carriage status was assessed months after the outbreak, and transient carriage is possible. Additionally, we were unable to identify published methods on how to assess *M. goodii* carriage.

*M. goodii* was not isolated from hospital A's water supply, based on swabs of biofilm and water from sinks, but other outbreaks of nontuberculous mycobacteria infections have been linked to municipal water (*9*). In one study, 95 (83%) of 115 dialysis centers had nontuberculous mycobacteria in their water supply (*10*). Many nontuberculous mycobacteria grow in biofilms that can form at faucet outlets, and several grow in hot, chlorinated, and distilled water (*9*). Although *M. goodii* was not isolated from any sink or water specimens, a water source could not definitively be ruled out. The hospital superheats the water system annually to 87.2°C (last done 1 month before this investigation) but does not culture water specimens. Therefore, water flora at the time of these infections could have been different from the flora present during the investigation. Additionally, because water specimens were collected with swabs rather than through a bulk water collection, *M. goodii* contamination might have been missed if concentrations were sufficiently low.

Although multidose vials were not linked to this outbreak, they caused other nontuberculous mycobacteria outbreaks (*9**,**11*). Several nontuberculous mycobacteria are resistant to mercury, which is commonly used as a preservative in multidose vials (*9*).

Nontuberculous mycobacteria are relatively resistant to disinfectants (*9*), and disinfectant and antiseptic effectiveness against *M. goodii* have not been reported. *M. smegmatis*, which is closely related to *M. goodii*, is sensitive to alkaline glutaraldehyde (*12**,**13*), povidone iodine (*13**,**14*), and chlorhexidene gluconate disinfectants. Sodium hypochlorite, ethanol, phenol, and quaternary ammonium are less effective (*13*).

*M. goodii* is generally susceptible to amikacin, ethambutol, and sulfamethoxazole. It has intermediate susceptibility to ciprofloxacin, doxycycline, and tobramycin; variable susceptibility to cefmetazole, cefoxitin, and clarithromycin; and resistance to isoniazid and rifampin (*1*). For mild wound infections, monotherapy with an oral agent for 4–6 months has been effective. In more severe disease, surgical debridement, initial combination therapy, followed by oral therapy, to complete 6 months of treatment has been effective (*2*).

The lack of an identified infection source made focused control measures difficult. Measures implemented by hospital A included requiring single-dose medication vials for invasive procedures; culturing for mycobacteria in wound cultures with negative bacterial growth; requiring staff to cover scrubs and remove shoe and hair covers when leaving the operating room area and to apply new covers upon reentering; requiring staff to use dedicated shoes in the operating room; having patients take preoperative showers with antiseptic soap; using tobramycin-impregnated cement in appropriate procedures; avoiding flash sterilization of implantable devices; and using phenol disinfectant weekly and quaternary ammonium disinfectant daily to disinfect operating rooms. Although data for *M. smegmatis* suggest that phenol and quaternary ammonium disinfectants might not be effective against *M. goodii* (*13*), evidence is insufficient to make firm recommendations.

Patients 2 and 3 received a diagnosis relatively quickly, but patient 1 had a prolonged, indolent infection and had an arthroplasty revision before diagnosis. Several wound cultures were performed without assessment for mycobacterial infection. Although documented (*9*), nontuberculous mycobacteria wound infections are less common than bacterial infections, and clinicians might be less aware of nontuberculous mycobacteria as potential causes of surgical site infections.

Research is needed to determine effective antiseptics and disinfectants against *M. goodii*. Clinicians should be aware of the association of nontuberculous mycobacteria with surgical site infections and consider testing for mycobacteria when a Gram stain shows notable numbers of leukocytes, but wound cultures are negative for bacteria.
